# Perceptions of Research Bronchoscopy in Malawian Adults with Pulmonary Tuberculosis: A Cross-Sectional Study

**DOI:** 10.1371/journal.pone.0165734

**Published:** 2016-10-28

**Authors:** Andrew D. McCallum, Deborah Nyirenda, Wezzie Lora, Saye H. Khoo, Derek J. Sloan, Henry C. Mwandumba, Nicola Desmond, Geraint R. Davies

**Affiliations:** 1 Malawi-Liverpool-Wellcome Trust Clinical Research Programme, College of Medicine, University of Malawi, Blantyre, Malawi; 2 Institute of Translational Medicine, University of Liverpool, Liverpool, United Kingdom; 3 Institute of Infection and Global Health, University of Liverpool, Liverpool, United Kingdom; 4 Liverpool School of Tropical Medicine, Liverpool, United Kingdom; 5 Liverpool Heart and Chest Hospital, Liverpool, United Kingdom; University of Padova, Medical School, ITALY

## Abstract

Bronchoscopy is an established research tool in Malawi, enabling collection of pulmonary samples for immunological, pharmacological, and microbiological studies. It is, however, an invasive clinical procedure that offers no direct benefit to volunteering participants when used in a research capacity alone, and thus informed consent is essential. This study aimed to explore TB patients’ understanding of research bronchoscopy, what would motivate them to participate in research bronchoscopy, and their concerns, in order to inform consenting processes for future clinical studies. We used a qualitative research design. Two focus group discussions were conducted with community members and TB patients to understand their perceptions of bronchoscopy. Transcripts were coded by multiple co-authors and thematic content analysis was used to analyse main findings. We found that Malawian patients with pulmonary TB were willing to participate in a study using research bronchoscopy for health assessment and access to improved healthcare. We identified information of value to potential participants when consenting to that may lessen some of the anxieties expressed by participants. Patient and public involvement is essential to improve informed consent and institutional trust.

## Introduction

Fibreoptic bronchoscopy is well-established in routine clinical practice for diagnostic and therapeutic purposes; with bronchoalveolar lavage (BAL) internationally accepted as a research tool [1]. Our research programme in Malawi has used research bronchoscopy to collect pulmonary samples for immunological and microbiological studies, and plans to extend its’ use to assessment of intrapulmonary pharmacology, specifically for tuberculosis (TB) drugs.

While bronchoscopy for research purposes has previously been done in healthy adult volunteers and sputum smear-negative pulmonary TB suspects, there is no precedence in Malawian adults with microbiologically-confirmed sputum smear-positive pulmonary TB. Bronchoscopy is an invasive clinical procedure that offers no direct benefit to volunteering participants when used in a research capacity alone. Complications are rare but include chest pain, fever, pneumonia, vocal cord injury, epistaxis, and extremely rarely, death, and thus informed consent is essential [2–4]. Previous work from Malawi suggested that the majority of bronchoscopy-experienced volunteer research participants reported that they valued the research study they were involved in, while new volunteers expected to benefit directly from the research [2]. The main perceived benefits were health assessment and prompt treatment if unwell, rather than remuneration. While most research participants felt they received adequate information before the study, semi-structured interviews identified limited understanding of the procedure in some participants. In order to inform the design of our future studies in TB patients, we sought to involve potential participants in the development of our consenting process and ensure that we addressed their concerns in advance of any procedure.

The Declaration of Helsinki outlines the ethical principles of medical research, and enshrines the need for informed consent, freedom from coercion, and protection of patient safety in research studies [5]. Health literacy, trust, and the quality of the interaction between patient and provider may all positively influence the quality of the consenting process and extent of engagement in research [6]. While written information and signed consent represent the standard, difficulties and controversy may arise in low-income settings where low levels of formal education, limited access to medical services and differing values and interpretation of health and illness make the practical application of these standards challenging [7, 8]. Engaging patients or public in research prior to study implementation is therefore seen to address their concerns, enhance trust, and can improve informed consent, mutual learning, recruitment and retention of research participants [9]. Patient and public involvement (PPI) is increasingly being advocated in health research to prevent harm to participants, improve the relevance of information sheets and the quality of informed consent [10, 11]. Boulanger (2013) however argues that little attention has been given to community engagement or PPI in TB research and this paper contributes to this knowledge gap [12].

We aimed to explore the needs and concerns of prospective research bronchoscopy participants, in order to inform study design, information provision, and consenting processes for our future clinical studies. This descriptive study highlights topics of importance to participants in research bronchoscopy studies, and may inform consenting processes for other invasive procedures in low-income settings.

## Materials and Methods

### Research team

Focus group discussions (FGDs) were facilitated by two experienced female, Malawian Social Scientists (DN and ML). At the time of data collection, DN was a Social Science PhD candidate and ML was a Social Science Researcher, both based at the Malawi-Liverpool-Wellcome Trust Clinical Research Programme. Both were native Chichewa speakers. The principal investigator (AM) is a British Infectious Diseases Physician who was planning to use bronchoscopy in a study assessing the intrapulmonary pharmacology of TB drugs. He was present at FGDs to answer any study- or bronchoscopy-specific enquiries, but as a non-Chichewa speaker, his role was limited to that of an observer.

Study participants had not met the researchers prior to taking part in the FGDs. Study researchers were not part of normal TB service provision, and participants were advised that taking part would not affect their current care. Participants were aware that AM was planning a bronchoscopy-based study of TB drugs, and that this work would be used to guide the recruitment and consenting processes in future work.

### Study design

This was a cross-sectional study of adult patients with pulmonary TB, and other members of the community in urban Blantyre, Malawi. We used a phenomenological approach to gather information on the perspectives and views of TB patients with regards to research bronchoscopy studies. Two FGDs were organised with the assistance of the TB officers at Queen Elizabeth Central Hospital (QECH), a large district and referral hospital. The first group (FGD1) consisted of mixed community members with some experience of TB—for example, spouses / carers of patients. The second group (FGD2) consisted of TB patients on treatment, but living at home and referring regularly to hospital. This enabled comparison of the perception of research bronchoscopy between healthy lay persons and TB patients. While many of the participants had experience of TB illness and care, none had experience of bronchoscopy. FGDs were chosen to enable the exploration of abstract concepts and social norms in the absence of embodied experience of bronchoscopy in these groups.

The two FGDs occurred in Ndirande, an urban settlement in Blantyre in the catchment area for QECH, in July 2014. This site was chosen as a future recruitment location for research bronchoscopy studies. Participants were included provided they were over 18 years old and could give informed consent. TB patients registered and receiving treatment at Ndirande Health Centre—prospective participants to our future work—were invited to attend a FGD. Twelve participants were recruited into each group, in approximately equal numbers of men and women. Participants were recruited by convenience sampling, reflecting the process of recruitment to the proposed research bronchoscopy study. By interviewing mixed groups, we aimed to capture the dynamic in the TB Registry, where mixed participants registering for TB treatment would be recruited to a bronchoscopy-based study. Three participants in FGD2 failed to attend the FGD. The demographics of the participants are summarised in S1 Table. All of the FGD participants were employed and educated to a minimum of Standard Two (primary school). Twelve participants were educated to secondary level. The FGDs occurred in a community hall.

Participants were provided with an information sheet for the study in Chichewa, the native language of the participants. After written consent to participate, the information sheet explaining the proposed research bronchoscopy TB pharmacology project (S1 File) was read to the participants in Chichewa, and they were given the opportunity to ask questions. Sections requiring further clarification, such as the details of the bronchoscopy procedure and the difference between research and clinical care, were explained further and the relevant sections re-read. The content of the information sheet was refined for FGD2 based on the feedback from the first group. A topic guide with open-ended questions was used to facilitate the FGDs (S2 Table), and included the broad themes of understanding of research, motivation for participation in studies of this nature, concerns, and feedback. These themes were developed through a deductive conceptual framework, drawing on pre-conceptions and previous work on bronchoscopy-based research in Malawi [2]. FGDs were recorded using a digital recorder, transcribed verbatim and translated into English. FGDs took approximately two hours, with field notes taken during the FGDs. Transcripts were checked for quality and accuracy by those who conducted the FGDs

### Analysis

The FGD transcripts were coded manually by AM, DN, and WL. We developed an initial coding framework based on a combination of research questions and an inductive approach to document emergent themes from the data. Emergent themes were triangulated through multiple and independent coding (AM, DN & WL). The coding frameworks were compared, refined and updated through regular meetings. Using thematic content analysis, a matrix of responses from the FGDs was generated in Excel (Microsoft Excel 2013, Microsoft Corporation, Redmond, WA, United States of America). The responses from the two FGDs were compared for similarities and differences across each theme using constant comparison approaches [13]. Direct quotations from research participants cited were chosen explicitly to represent dominant themes emerging from the thematic analysis. We drew on COREQ guidelines when reporting our findings.

### Ethical approval

This study was approved by the University of Malawi, College of Medicine Research Ethics Committee (approval number: P.05/14/1575). All participants gave written informed consent (or a witnessed thumb print if illiterate) to take part in the study.

## Results

### Understanding of health research

In both FGDs, participants had difficulty distinguishing between research and a health intervention. Both groups expressed a motivation to participate in this proposed project as they expected to benefit directly from clinical assessment. For some, this was simply the benefit of being a part of a research study with clinical input beyond that received in standard TB care. For many participants however, they felt that the bronchoscopy would give them immediate information about their “*health status”*, and an indication of how well the TB drugs were working in their body. A typical response was:

“I can join this research study because I would like them to examine me and find out how I am inside (internally) as I am taking the drug, saying: is the drug functioning well? On the other hand, there is a possibility that they can add more of that drug to me.”

***Participant, FGD1***

In FGD2, the interviewers attempted to improve the informed consent process by carefully explaining the difference between research and clinical care, and that participation in health research did not equate to receiving *“special treatment”*. The patient information sheet was adjusted to state that participants would not benefit directly from participation in the study, but that the results may lead to improved treatments for TB in the future. Despite these changes, those in FGD2 still expected immediate benefit from the research in terms of improved therapy or individual feedback on drug effectiveness.

### Motivation for participation

Participants were invited to consider their motivation for participating in a research bronchoscopy study in which they did not personally stand to benefit. Motivation was typically framed as altruistic by participants in both FGDs, in that it may benefit future patients with TB. One participant, an older male, gave the analogy:

***Participant*:** “*We all seem to be those people helping to build a bridge*, *by assisting to pass over the materials required to construct the bridge*, *so that every person should what*?*”*

***All*: (*overlap*)** “*they should cross properly*”

***Participant*:** *“Therefore we are not forced; it is all up to you to decide for yourself saying*: *for the bridge to be complete there is need for me to go and cut down trees*.”

***Participant, FGD1***

Some reported they had a responsibility to participate in TB research to further research and improve care.

“… research is done to find an answer. Therefore, for the answer to be found quickly, there is need for me to be with them on the same side [I need to participate in the research] so that the research should move on well.”

***Participant, FGD1***

Those with TB in FGD2 discussed the burden of TB, and that by participating in research studies such as this they could help fight to eliminate the disease.

### Concerns

#### Fear of exploitation

Participants in FGD1 raised concerns that research may be exploitative, and that safeguarding of participant’s safety may be a secondary concern. Typical responses included:

“On the other hand they say: whenever a research is coming up, the government indicates or even signs that it doesn’t care for those 200 or 300 people [enrolled in research], even if they die. This is the reason why most people get discouraged saying: aah should I die (laughter), the fears happen to be there.”

***Participant, FGD1***

“People say: whenever there is a research, we feel that maybe the health sector [Ministry of Health] would like to use us to gain knowledge. Most people like to say that: aah I cannot join a research; they would like to learn from our bodies, most people like to put it that way.”

***Participant, FGD1***

“When new research projects are coming in, some people say: they want to learn using our bodies, they are the people chewing a lot of money, but when the research is happening, it is done on us.”

***Participant, FGD1***

This fear of exploitation was not expressed in FGD2 –those with personal experience of TB illness and healthcare delivery.

#### Fear of potential risks of research bronchoscopy

Having explained the research bronchoscopy patient information sheet to the focus group participants, they were invited to give their opinions on the use of bronchoscopy as a tool to investigate TB drugs. Participants were mainly in favour of the research, though some expressed some apprehensions. Much of the fear emerged from participants having a limited understanding of the bronchoscopy procedure and any potential risks given the information provided. Anxieties stemmed from being unable to visualise the bronchoscope itself, and were assuaged by inclusion of an illustration and explanation of the size of the instrument in FGD2.

In FGD2, a number of participants sought clarification on the ‘fluids’ taken during the bronchoscopy procedure. It became apparent that the use of BAL, or washing, was not clear, and that participants took this process to be akin to taking blood (by venepuncture). As such, a number of participants discussed the bronchoscope entering *“small veins”* within the lungs. Participants sought clarity on the appearance and texture of the bronchoscope, concerned it may be the size of a *“water pipe”* or made up of metal. Another participant in FGD1 expressed anxieties that the bronchoscopy procedure may adversely affect the circulation of the blood if they draw a lot of fluid from one’s body.

We sought comments on further information of importance to potential participants. Participants requested specific detail about the duration of the intervention, and the expected duration of any side effects. Fever was included as a rare side effect in the patient information sheet (S1 File), but a number of participants in FGD1 focussed on how long this side effect could be expected to last. By including this information, they would have an idea when to be worried about the duration of side effects and seek help. Rather than deterring participants, full disclosure of risks was appreciated. Furthermore, accurate information was important for trust of the study team.

**"***As for me*, *I feel that in that case*, *if I feel pain or whatsoever [side effect]*, *the way they have explained it*, *and they have counselled me that you will feel pain to this extent*, *if I experience it*, *I will come back [for a second bronchoscopy]*. *But if it [the side effect] exceeds the limit [beyond what was initially discussed]*: *like you said*: ***24 hours*** *of pain and fever for* ***two days***, *this means that the research study is different from what you have told us*.*”*

***Participant, FGD1***

Other information that would be appreciated included how long the cough may last after bronchoscopy, and how long the anaesthesia would be expected to last. Pain and bleeding were rarely mentioned in the discussions, but again, duration and amount were issues that concerned participants.

#### Views on HIV testing

Routine testing for HIV as a criterion for participant recruitment was raised as a concern by participants in FGD1, and there was a feeling that this should be optional.

“As for me, what I feel is that most of us get troubled inside our hearts, saying: AIDS is incurable, therefore people become scared, that it’s better to stay without knowing your health (HIV) status. But as for TB we know that this is curable.”

***Participant, FGD1***

This did not seem to be an issue with the TB group—FGD2 –presumably as HIV testing and counselling is an established part of the TB diagnostic process and reflected their clinical experience. This was echoed by a number of participants, who appreciated the importance of diagnosis, even when asymptomatic, primarily for treatment reasons:

**Interviewer:** “…Is the person joining this research study supposed to undergo an HIV test?”

**All:** “Yes”

**Interviewer:** “Do you feel this is appropriate?”

**All:** “Yes!”

**Interviewer:** “Why is that so?”

**Participant:** “Because he/she will know about her body if he/she has TB…therefore you are in a position to know whether you have TB as well as HIV… and you get treated for both of these”

***Participants, FGD2***

### Results feedback and compensation

A common concern of participants was that results should be fed back to the community, with some commenting that they do not even know when a study is ended:

“When the research comes to an end, they just leave and go… We still think that we are enrolled in the research study not knowing that we are out.” (laughter)

***Participant, FGD1***

As payment for participating, information about the study’s results was considered to be a requirement:

“…because I was one of those people participating in the study [research participant], therefore I should know the answer to the research study [research findings].”

***Participant, FGD1***

Other than the results of the study, an idea of how many people the study results stand to benefit was requested, and whether any recommendations were made to improve TB treatment by the researchers.

Some participants felt that the clinical assessment itself was a motivation and reward for recruitment into a research bronchoscopy study, and many voiced that any adverse events or intercurrent illnesses occurring within the course of such a study should be treated promptly. Participants in FGDs with TB patients, were aware of other studies providing prompt healthcare outside the study interventions. One participant in FGD2 indicated that they felt that medical care above that available in Malawi may be a suitable recompense for an adverse event, but this opinion was not echoed by the other participants.

“For instance if a person has severe bleeding and not as expected, saying: it did not come out the way you had expected it to come out, you must give her treatment to stop the bleeding. They must take her to big hospitals if they feel that the problem is major, regardless of how much it will cost…the researchers must take responsibility if the patient becomes severely ill because they enrolled in the research…They should find treatment even if it means flying the patient outside the country.”

***Participant, FGD2***

Compensation requests were modest: reimbursement for transport or a meal allowance were the commonest suggestions. Others suggested that counselling and additional study visits were of benefit enough to them. In FGD1, there were a number of participants who felt that the compensation should reflect the distance travelled to the hospital, and furthermore, that consideration should be given to lost earnings as a result of taking part:

“Because from a women's perspective, if you want us to be in a research study we will have to leave our homes at the time that you have asked us to meet you. Maybe we had plans to go and pick sweet potato leaves [for food], we failed, because we only have limited time. With the money [compensation] given we could pass by the market and use the same money to buy some mustard and charcoal, and cook [a meal].”

***Participant, FGD1***

## Discussion

In this study, we explored perceptions of research bronchoscopy in a population of Malawian adults, in order to inform the design of future clinical studies using bronchoscopy. Findings indicate a general willingness to participate in studies of this nature, primarily for accrual of personal benefits. Participants expected to benefit directly from clinical assessment, and had difficulty distinguishing between research and routine clinical care. We identified shortcomings in the informed consent process that will inform clinical practice for future research bronchoscopy-based studies. Through the FGDs, we identified information of value to potential research participants that will guide subsequent engagement strategies.

In this group of bronchoscopy-naïve individuals, there was a common difficulty in distinguishing between interventions performed solely for research purposes, and standard clinical care. Many participants expressed a wish to participate in a research bronchoscopy study for assessment of their *“health status”*, and expected to receive immediate feedback on drug efficacy and their response to treatment. This was particularly true in FGD1, where participants expected individualised feedback post-bronchoscopy rather than aggregate data at study close. This confusion has been described as ‘therapeutic misconception’ in similar research settings—a belief that every aspect of a research project was designed to benefit the individual directly—and raises concerns that participants may have difficulty distinguishing between investigational research and health intervention when making an informed choice [6, 14]. Rather than being a fault of the participant, we would argue that this confusion was a shortcoming of the informed consent process and our explanations to FGD participants.

The model of therapeutic misconception places the blame for the misunderstanding with the study participants, and has been described as particularly problematic in resource-limited settings due to a combination of low levels of literacy, poor education, poor access to healthcare, high disease burden, and indeed the impact of illness, suffering, poverty, and provider roles on decision making [14–16]. A meta-analysis of informed consent comprehension in African settings reported that comprehension of key concepts was poor [15]. Understanding the investigative or experimental nature of research is clearly a key component in collecting true informed consent, but may be more challenging in a resource-limited setting. Firstly, care quality in Malawi is limited by shortages of healthcare personnel and medications in Government facilities [17]. Researchers have an ethical and moral obligation to provide healthcare to benefit participants, and thus the appropriate provision of substantial and quality medical care in research can result in genuine difficulty in distinguishing between healthcare and research [6, 17]. Rather than a misconception, the provision of healthcare to study participants may provide incentive to recruitment, with participants actively choosing to enrol in research as a rational endeavour to access better-resourced care. Secondly, the onus should be on the researcher to properly inform potential participants of the research nature of the study, and to underscore the assertion that there can be no guarantee of therapeutic benefit [18]. While we attempted to emphasise the investigational nature of the research in FGD2, participants still expected to directly benefit from bronchoscopy. This information will be used when planning our engagement strategy and informed consent processes in future work.

By inviting bronchoscopy-naïve individuals to the FGDs, we aimed to capture a group similar to those that would be recruited to a future bronchoscopy-based study, and gather their views of research bronchoscopy in the absence of embodied experience. As such, participants were asked to consider their motivation in an abstract way. Both FGDs framed their motivation for participation as altruistic: that it would benefit future patients with TB, or that taking part would contribute to eliminating TB disease. This is likely to reflect a social desirability bias: a product of the need to impress the group rather than a true individual perspective [19, 20]. When asked later to consider compensation for participation in research, participants stated that clinical assessment was both a motivation and a reward, and that they expected to be treated promptly in the event of adverse events or intercurrent illnesses. Furthermore, they should stand to benefit should the study lead to improved TB treatment. Rather than altruism, participants were motivated by eventual self-benefit, or the belief that they may lose out by non-participation.

Limited data from resource-poor settings exists on the motivation of individuals to participate in non-therapeutic biomedical research. In India, one study reported that healthy individuals were most likely to cite financial reward as their motivation, whereas patients would participate after being invited by their treating physician [21]. Altruism, free medical check-up, curiosity, and personal health benefit were also cited as motivation for recruitment to non-therapeutic trials [21]. Recently, a qualitative study from Blantyre, Malawi, identified that ancillary care, monetary and material incentives, and more thorough medical diagnosis encouraged participation in biomedical research [17]. While improved medical care was cited in this study, monetary and material incentives were mentioned rarely, and when compensation was discussed, the requests of participants were relatively modest. Participants in FGD2 commented that compensation should consider lost earnings due to the time spent participating in research. These costs may not be routinely considered in study design, and risk participants being under-compensated rather than induced for their role in research [22]. Both groups expected feedback of results at study completion as compensation for taking part. While one FGD2 participant stated that they may never know if the study they participated in has reached completion, care should be taken to provide community feedback because this important in encouraging institutional trust and ensuring ethical practice. Such feedback is already occurring at our research programme in Malawi through an existing Science Communication Department, through science cafes and community advisory groups, and researchers should continue to be encouraged to use them. Lack of feedback from research studies is a common complaint, even should those results be distressing [23–27]. This may simply be the provision of study conclusions to participants, but where appropriate, any results of importance to individual participants should be fed back to enable appropriate medical care. This has been highlighted in genetic studies, where data on future health risks may be uncovered, but could equally be applied to incidental findings during bronchoscopy [28, 29]. Ultimately, studies should be reciprocal, and not simply extractive.

Whereas previous work from Malawi interviewed bronchoscopy-experienced individuals [2], we sought to assess the understanding of research bronchoscopy in bronchoscopy-naïve participants after provision of a patient information sheet. Potential participants understandably had difficulty envisioning a bronchoscope, and requested information on its’ dimensions, appearance, and texture: information that we included for FGD2. Given that these participants were bronchoscopy-naïve, and asked to imagine the procedure, these misconceptions reflect shortcomings in the information provision by researchers, rather than a lack of participant education, and highlight areas for clarification in future bronchoscopy-based research. The FGD participants in this study were keen that the patient information sheet provided explicit information as to the duration and severity of any side-effects in advance of the study, and the accuracy of this information was important to fostering trust.

There are a number of limitations to this study that merit discussion. Firstly, none of these participants had prior experience of invasive procedures such as bronchoscopy, and were asked to consider their involvement in such research in an abstract manner. By so doing, we hoped to understand the perceptions and concerns of a bronchoscopy-naïve population to better reflect those individuals recruited into future projects. Secondly, the use of the FGD method likely shaped the responses to some questions, particularly around motivation. Participants will try to project a positive impression to the group, and the researchers, and should be taken into account in the interpretation of their comments. Furthermore, by choosing to participate in the FGDs in the first place these participants may already be biased in favour of research. It is difficult to know how individual experiences influenced responses with this method, and future work may consider how these responses translate into embodied experience in practice: how many would actually enrol in a research bronchoscopy study? In later work, we will invite TB patients undergoing research bronchoscopy to return for semi-structured interviews and focus group discussions to explore their experience-based perceptions of research bronchoscopy and the consenting process used.

Some of the study limitations were that only two FGDs were conducted, and we did not triangulate findings across different sources or methods. The small sample size reflected the availability of registered patients to attend the FGDs, and was felt to be adequate for a descriptive study to inform the design of a clinical study. We interviewed participants who were willing to attend the FGD, and supplemented this by interviewing relatives and carers. Nevertheless, the two FGDs gave diverse perspectives on bronchoscopy research and similar themes appeared in both groups. Three authors (AM, DN and WL) were also involved in the data analysis processes to enhance trustworthiness of our research findings.

In conclusion, we have shown that Malawian patients with pulmonary TB are willing to participate in a study using research bronchoscopy, and would do so for health assessment and access to improved healthcare. Clear, illustrated patient information sheets should help lessen some of the anxieties expressed by potential participants, and engagement with the community before and after the study is essential. This study contributes to Malawi-specific findings to issues around ethics and informed consent for invasive procedures that will be of interest to a wider audience.

## Supporting Information

S1 File Draft Patient Information Sheet. (DOCX)

S1 Table Subject Demographics. (DOCX)

S2 Table Focus Group Discussion Topic Guide. (DOCX)
