# Supplementary material for: Perceptions of Research Bronchoscopy in Malawian Adults with Pulmonary Tuberculosis: A Cross-Sectional Study
Source: PLoS One. 2016 Oct 28;11(10):e0165734. doi: 10.1371/journal.pone.0165734 (PMC5085028; doi:10.1371/journal.pone.0165734)
Supplement: S1 File — (DOCX) [file pone.0165734.s001.docx]

Draft Patient Information Sheet

### Proposed Research Bronchoscopy TB Pharmacology Project

Please note, you are NOT being asked to participate in this study – this is a project we would like to perform in the future. We would like to give you the information about the study, and ask for your opinions and any concerns about the information we present.

### Introduction

You are being invited to take part in a research study. Before you decide it is important for you to understand why the research is being done and what it will involve. Please take time to read the following information carefully and discuss it with others if you wish. Ask us if there is anything that is not clear or if you would like more information. Take time to decide whether or not you wish to take part.

### What is the purpose of this study?

Tuberculosis (TB) is an infection which occurs frequently in Malawi. Despite the best available treatments, some people still become very ill with this infection and some die. Treatment for TB takes a long time – a minimum of six months. Little is known about how the medicines work in the lung to fight the infection, or why in some people the medicines stop working and the TB becomes resistant. This study would look at how well the medicines get to the lung. Hopefully this will guide us in using the medications better, in working towards shorter or more effective treatments.

### Why have I been chosen?

You have been chosen because the doctors looking after you have decided to start treatment for TB.

### Do I have to take part?

It is up to you to decide whether or not to take part. If you do decide to take part you will be given this information sheet to keep and be asked to sign a consent form. You will still be free to withdraw at any time and without giving a reason. A decision to withdraw at any time, or a decision not to take part, will not affect the standard of care you receive.

### What will happen to me if I take part?

After you agree to take part in this study, you will be seen by a member of the research team. You will be asked some more questions about your health, and examined clinically. Then some blood will be taken this will be about 30-50ml (3-5 tablespoons) of blood. These blood tests will look at your liver, kidneys and blood count; some more specialised tests will be performed to look at your immune system in detail.

One important thing which affects how well a person responds to treatment for TB is HIV infection. We will need to know whether you have HIV infection. After you start TB treatment, somebody will speak to you in more detail about a blood test for HIV and what this involves. You can choose not to have this test, but if that is the case, we will not be able to include you in the study. When we test for HIV, the result remains confidential. This means that people will not be able to find out the result unless you choose to tell them. The blood samples we take from you will be coded, and so nobody looking at them will know they belong to you.

After you have been on treatment for 6-8 weeks, you will be asked to return to the hospital for the day. You will take your TB medications in the morning, and blood samples (about 3-5 tablespoons of blood) will be taken at the time of your TB medications, and 2, 4, 6 and 8 hours after the medication. This will allow us to work out the level of TB medicine in your blood. You will be examined clinically to assess your health. You will be asked not to eat or drink on the morning of coming into hospital. You will be asked to sign a form giving permission to take part in the study.

Between blood tests, you will be asked to have a bronchoscopy. This is a test that allows us to look inside the lungs with a short, thin tube with a mini camera in the tip called a ‘bronchoscope’. You will be asked to sit on a trolley and bite on a plastic mouth guard. A probe will be attached to your finger in order to record your oxygen levels and pulse rate during the procedure. The tube is passed through your mouth, past your vocal cords (voicebox), into your windpipe and into the airways. Fluid (about 1 cupful) is put into a single small air tube in the lung then sucked back up into a specimen jar. This collects cells from the air sacs of the lung for testing in the laboratory. This may cause you to cough a little during the procedure. You will be given a spray to numb the back of the throat, and some medication to calm you down should you wish it. The procedure usually lasts 20-30 minutes.

### What are the side-effects/ risks of taking part?

You will not receive any drugs or treatment other than those your doctor has recommended. You will need to have some blood taken – there may be a small amount of pain and bruising at the site of needle puncture for a short time.

The bronchoscopy procedure may be uncomfortable, but it is not usually painful. You may cough from time to time during the procedure. There are few risks of having the procedure. The procedure will be performed by a skilled operator with a doctor present for safety.

Common risks and complications (more than 5%). These symptoms usually settle within a day:

- Sore throat
- Sore nose
- Cough
- Coughing up blood (haemoptysis)
- Low oxygen levels. You will be given oxygen.

Uncommon risks and complications (1-5%):

- Collapsed lung. This is rare as we will not be taking a sample of tissue – a biopsy. If this happens, you may require a longer stay in hospital with a tube inserted between the ribs to reinflate the lung.
- Heart problems. A brief minor strain may be put on the heart. This can cause abnormal beating of the heart. It rarely causes fluid to collect in the lungs or a heart attack.
- Asthma-like reactions. The air tubes can be narrowed due to irritation by the procedure. This is usually treated with asthma medication.

Rare risks and complications (less than 1%):

- Narrowing of vocal cords (Laryngospasm). This is usually brief and rarely a problem.
- Fever. This is treated with paracetamol (Panadol).
- Death as a result of this procedure is rare.

Your treatment will not be delayed or affected by your taking part in this study. We will require you to attend the hospital once more during the first two months of tuberculosis treatment than you would if you were not taking part in the study. We will pay for your travel expenses. As the procedure can be unpleasant, you will be given compensation for the inconvenience of taking part in the study.

You will be invited to return 2 days after the procedure to check on your health.

### What are the potential benefits of taking part?

There are unlikely to be any direct benefits to you in taking part in this study. But we hope our results will lead to improved treatments for tuberculosis.

### What if something goes wrong?

If you wish to complain about any aspect of the way you have been approached or treated during the course of this study, complaints should be made via the College of Medicine.

### Will my taking part in this study be kept confidential?

Confidentiality will be maintained at all times. All samples will be coded (but it will be possible to link results to anonymised data collected from this study). We will combine all the results from this study and present the data on groups of patients (rather than individual patients). No individual will be identifiable from any presentation or publication that ensues from this research.

### What will happen to the results of the study?

We will combine all the results from the patients taking part in the study, and discuss important results at scientific meetings. We will present the results in scientific publications and presentations.

### What happens when the study is finished?

After the study is complete, you will complete your treatment for tuberculosis as planned.
