# Supplementary material for: Perceptions of Research Bronchoscopy in Malawian Adults with Pulmonary Tuberculosis: A Cross-Sectional Study
Source: PLoS One. 2016 Oct 28;11(10):e0165734. doi: 10.1371/journal.pone.0165734 (PMC5085028; doi:10.1371/journal.pone.0165734)
Supplement: S1 Table — (DOCX) [file pone.0165734.s002.docx]

Table 1

### Subject Demographics

| **DEMOGRAPHIC** | **FGD1** | **FGD2** |
| --- | --- | --- |
| **Number** | 12 | 9 |
| **Age (years)**  **18-29**  **30-49**  **50-75**  **>75** | 2 (16.7%)  10 (83.3%)  0 (0%)  0 (0%) | 3 (33.3%)  5 (55.6%)  1 (11.1%)  0 (0%) |
| **Gender**  **Male**  **Female** | 5 (41.7%)  7 (58.3%) | 5 (55.6%)  4 (44.4%) |
