# Supplementary material for: Perceptions of Research Bronchoscopy in Malawian Adults with Pulmonary Tuberculosis: A Cross-Sectional Study
Source: PLoS One. 2016 Oct 28;11(10):e0165734. doi: 10.1371/journal.pone.0165734 (PMC5085028; doi:10.1371/journal.pone.0165734)
Supplement: S2 Table — (DOCX) [file pone.0165734.s003.docx]

Table 2

### Focus Group Discussion Topic Guide

| **Focus Group Discussion Topic Guide** |
| --- |
| What are your views of this type of project? |
| Would you be interested in participating in this kind of project?  What would motivate you to participate?  What would make you reluctant to participate? |
| What information would you like beforehand? |
| [Having read out patient information sheet]  What information do you think is relevant?  What information have we missed out?  Did we give enough information about risks / dangers? |
| What feedback, if any, would you expect after the study? |
| Would you find HIV testing acceptable as a requirement for the study? Why? |
| What compensation would you deem adequate? |
